# Ripple effects in a communication network: anti-eavesdropper defence elicits elaborated sexual signals in rival males

**DOI:** 10.1098/rspb.2023.1910

**Published:** 2023-12-20

**Authors:** Brian C. Leavell, Hoover Pantoja-Sánchez, Viviana Vélez, Claire T. Hemingway, Kyle Wilhite, Wouter Halfwerk, Ximena E. Bernal

**Affiliations:** ^1^ Department of Biological Sciences, Purdue University, West Lafayette, IN, USA; ^2^ Department of Electronic Engineering, University of Antioquia, Medellín, Antioquia, Colombia; ^3^ Program of Study and Control of Tropical Diseases, University of Antioquia, Medellín, Antioquia, Colombia; ^4^ Department of Ecology & Evolutionary Biology and Department of Psychology, University of Tennessee, Knoxville, TN, USA; ^5^ Department of Integrative Biology, University of Texas, Austin, TX, USA; ^6^ Department of Ecological Science, VU University, Amsterdam, The Netherlands; ^7^ Smithsonian Tropical Research Institute, Apartado, Balboa, Ancón, Panamá

**Keywords:** communication, foraging, male–male competition, predator–prey, vibratory cue, sexual selection

## Abstract

Emitting conspicuous signals into the environment to attract mates comes with the increased risk of interception by eavesdropping enemies. As a defence, a commonly described strategy is for signallers to group together in leks, diluting each individual's risk. Lekking systems are often highly social settings in which competing males dynamically alter their signalling behaviour to attract mates. Thus, signalling at the lek requires navigating fluctuations in risk, competition and reproductive opportunities. Here, we investigate how behavioural defence strategies directed at an eavesdropping enemy have cascading effects across the communication network. We investigated these behaviours in the túngara frog (*Engystomops pustulosus*), examining how a calling male's swatting defence directed at frog-biting midges indirectly affects the calling behaviour of his rival. We found that the rival responds to swat-induced water ripples by increasing his call rate and complexity. Then, performing phonotaxis experiments, we found that eavesdropping fringe-lipped bats (*Trachops cirrhosus*) do not exhibit a preference for a swatting male compared to his rival, but females strongly prefer the rival male. Defences to minimize attacks from eavesdroppers thus shift the mate competition landscape in favour of rival males. By modulating the attractiveness of signalling prey to female receivers, we posit that eavesdropping micropredators likely have an unappreciated impact on the ecology and evolution of sexual communication systems.

## 1. Introduction

Eavesdropping enemies can exert intense selective pressure on their prey, particularly in sexual communication systems in which mating signals directly influence access to mating opportunities [1,2]. In response, signalling prey evolve defensive strategies to reduce predation risk, such as counter-attacks, evasive behaviours and private signals [3–7]. Despite the abundant evidence that signals and signalling strategies have been shaped by selective forces imposed by eavesdropping enemies [2], to what degree risk from these enemies extends beyond the signaller remains an open question. Initial findings suggest that conspicuous signals can increase the risk of attack for a signaller's target receiver [8] and nearby signalling heterospecifics [9]. The influence of eavesdropping enemies may also extend to higher levels of organization by driving the stability and structure of social interactions [10,11].

The aggregation of signallers in leks is hypothesized to be a defensive strategy that reduces an individual signaller's risk of attack (i.e. the ‘dilution effect' [12,13]). Yet, given that mating aggregations are also social environments in which signallers attend and respond to each other's behaviours, eavesdropping enemies have the potential to induce cascading effects across prey social interactions by modulating their prey's behaviour. Signallers in lek mating systems (typically males) gauge the level of competition from nearby rivals via their perception of rival signal elaboration and spatial proximity [14]. Males that can modify their signals dynamically adjust their signal elaboration based on the perceived local competition intensity [15,16]. Signallers also modify their displays directly in response to perceived eavesdropper risk [17,18] or indirectly via a trade-off with anti-eavesdropper behavioural defences [19]. As a consequence of influencing their prey's signals and cues, eavesdropping enemies influence the stimuli used by neighbouring rivals to assess local competition and update their own signalling strategy. In leks, we can thus expect that eavesdropper attacks indirectly result in changes in signal elaboration in rival signallers.

Such shifts in signalling behaviour during competition between males can modulate the costs and benefits associated with signalling for individual signallers. Given that relative signal attractiveness of competing males affects female mating decisions, eavesdroppers may modulate reproductive opportunities throughout the lek. Similarly, eavesdropper-induced changes in signal elaboration in a signaller and his rivals may impact foraging decisions by other eavesdropping predators, thereby modifying each signaller's relative predation risk. As eavesdropping enemies may directly and indirectly influence each other through their effects on the signaller, different types can impose diverse effects. Indeed, eavesdropping enemies vary greatly in their consumer strategies and thus the risks they impose. For instance, predators consume multiple organisms, completely eliminating their resource's fitness, whereas micropredators consume resources from multiple organisms but do not individually eliminate their resource's fitness (*sensu* [20,21]). Here, we tested the hypothesis that eavesdropping micropredators modulate the relative attractiveness of interacting male competitors to mates and eavesdropping predators.

An ideal study system to examine the direct and indirect effects of eavesdropping enemies on mating signals is the túngara frog (*Engystomops pustulosus*). Males call as part of a chorus where they attend to the calls of up to 4 nearby males, and dynamically alter their call rates and complexity (i.e. number of ornamental ‘chucks’ appended to their call) in response to their neighbours' signalling strategies [22,23]. Increased call rate and complexity function, in part, as aggressive responses to nearby calling males [23,24]. Call rate and complexity are also positively correlated with attractiveness to females [25–28] and eavesdropping predators such as the fringe-lipped bat (*Trachops cirrhosus* [29]) and micropredators such as the frog-biting midges (Corethrellidae [30]).

In addition to the airborne acoustic component of a neighbour's mating call, male túngara frogs, which call while partially submerged in water, attend to incidental, call-induced water surface waves (hereafter ‘ripples'). They respond to the ripples of rival males, thought to be cues to assess competition intensity, by increasing their call rate and complexity [31–33]. Water ripples, however, are naturally elicited by a wide variety of sources. Thus, males face the ecological challenge of responding to useful stimuli (e.g. call-induced ripple from a competitor) given that similar stimuli also occur from sources associated with irrelevant (e.g. rain-induced ripples) or harmful conditions (e.g. predator-induced ripples).

Ripples are also frequently generated by calling túngara frogs that swat with their arms and legs to fend off attacks from eavesdropping, blood-sucking midges [30,34,35]. Consequently, these attacks limit the extent to which a male frog elaborates his signal and the degree to which competition affects his calling behaviour [19]. To examine the mechanisms underlying how eavesdroppers may affect the relative fitness of males within the social network by modulating the relative attractiveness to mates and risk to other eavesdropping predators, we investigated the vocal response of rival túngara frogs to swat-induced ripples. We predicted that, as with call-induced ripples, swat-induced ripples would elicit increased call rate and complexity in a nearby male competitor. If this prediction is supported, we predicted that the rival's calling response, compared to the limited call rate and complexity of a swatting male, would result in females and eavesdropping bats preferring the rival male. To examine these predictions, we tested responses of calling túngara frogs (*n* = 29) to simulated calls and ripples representing characteristics of these stimuli observed in wild frogs. Then, we assessed the preferences of female frogs (*n* = 24) and foraging bats (*n* = 5) between the calling behaviours of a swatting male and a neighbouring rival that responds to swat ripples. Ultimately, by unravelling the ecological mechanisms through which eavesdropping enemies may affect individual fitness outcomes throughout a social network, this work provides essential insights into the factors driving and constraining sexual selection and sexual signal evolution.

## 2. Methods

### (a) Rival male experiment

In August 2017, we collected calling male túngara frogs 1–4 h after sunset from breeding sites within 1.5 km of Smithsonian Tropical Research Institute facilities (Gamboa, Panamá; 9°07.0' N, 79°41.9' W). Frogs took part in behavioural experiments in a large outdoor flight cage (5 × 5 × 2.5 m) within 3 h following capture and before midnight. Following protocols established by the American Society of Ichthyologists and Herpetologists (https://asih.org/animal-care-guidelines), after each experiment, we weighed, measured and toe-clipped the frog (to prevent testing the same individual multiple times), and released him the following night at his collection location.

#### (i) Field recordings

To sample the natural variation of swat and call ripples from calling male túngara frogs, we recorded ripples from six males (*n* = 2 *in situ*; *n* = 4 in a planter tray filled with water from the breeding site, placed by the original calling site). We measured the frequency, velocity and displacement of the water ripples with a digital laser vibrometer (LDV; Polytech PDV-100; velocity = 20 mm s^−1^, low pass = 22 kHz, high pass = none). The laser was focused on a reflective marker floating on the water surface; its reflection is detected by the LDV, allowing measurement of the surface vibrational velocities. We recorded these measurements from the digital output of the LDV using a Marantz audio recorder (48 kHz sample rate, 24 bit). See electronic supplementary material for a full description of the recording setup. We analysed call ripples (52 samples from 6 males; range: min = 5, max = 13 per male) and swat ripples (66 samples from 6 males; range: min = 5, max = 15 per male) using the velocity measurements recorded from the LDV digital output. We also converted velocity data to displacement values for comparison with related studies that have used this variable to characterize call ripples in this species (e.g. [32,36]). All ripple characterization analyses were performed in Matlab R2016a (https://www.mathworks.com) using custom code [37].

The vertical velocity of a swat ripple differed depending on the location of the sensor relative to the side of the body that swats (electronic supplementary material, figure S1, and R scripts for details of the analysis). We therefore tested calling behaviour in response to both high- and low-velocity swat ripples to determine male responses to the range of swat-ripple intensities encountered in the wild. We used the point estimate of the median maximum velocity of all recorded swat ripples (5.84 mm s^−1^ peak-to-peak), which was between the 95% credible intervals for the median low- and high-intensity swat values, as a guide to calibrate low- and high-velocity swat playbacks. While there was variation across playbacks, the vast majority of calibration measurements were within the range measured from the field recordings (see electronic supplementary material, figures S2–3 and associated R scripts for further details [37]).

#### (ii) Experimental set-up

For our ripple playback files, we used a total of three field recordings. These files represented the median dominant frequency and median maximum velocity of either a (i) high-velocity swat, (ii) low-velocity swat or (iii) call ripple. As playback of call ripples always co-occurred with an airborne call playback, we ensured that the call ripple accurately coincided with the airborne call component by synchronizing their playback to match the temporal profile of the field recording. For the airborne component of the multimodal call playback, we generated a call comprised of a whine with one chuck, as this is the most common call complexity observed in natural settings (see electronic supplementary material for details) [38,39].

**Figure 1.**
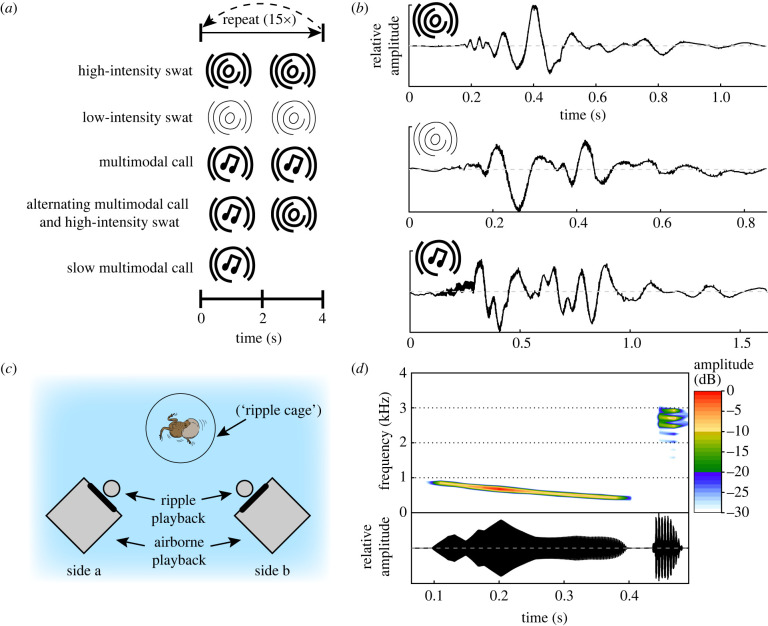
Playback stimuli and overview of experimental set-up. (*a*) Schematics of treatments presented to an individual male. (*b*) Oscillograms of the playback stimuli (normalized amplitude). From top to bottom: high-intensity swat ripple, low-intensity swat ripple, call-induced ripple. (*c*) Top-down view of experimental set-up (not to scale). Side used in experiment was randomized per trial to control for side biases. (*d*) Spectrogram and oscillogram of airborne call (whine with one appended chuck) that was synchronized with the call-induced ripple for the multimodal call stimulus. Colour gradient depicts relative sound intensities.

Prior to an experiment, a male (*n* = 29 frogs; no males were from field recordings) was placed in a pool (2.6 × 2.1 m) of dechlorinated tap water. The male was contained within a ripple cage but could otherwise call freely. This ripple cage, used in a previous study of call-induced ripples, is acoustically transparent to low-frequency ripples [33]. The cylindrical ripple cage (10.5 cm total diameter) consisted of a plastic, circular base and top held in place by three evenly spaced, vertical screws (approx. 0.4 cm diam.). Monofilament fishing line (approx. 0.36 mm thick) was strung vertically, encircling all sides of the plastic base and top (spaced approx. 0.25 cm apart creating an acoustically transparent cylinder 8 cm in diameter) to prevent the frog from escaping. Once the male was consistently calling, he was presented with the first of four trials, which were presented in random order and separated by at least 1 min of silence. The order of presentation did not affect male responses (electronic supplementary material, tables S1 and S2). Each trial consisted of a 1min silent control playback and 1minute playback of one of the following treatments: (i) high-intensity swats, (ii) low-intensity swats, (iii) multimodal calls (airborne calls and call ripples) and (iv) multimodal calls alternating with high-intensity swats (figure 1). Each stimulus was played back every 2 s, which is consistent with the average natural call rate [38] and within the natural range of swat rates [19]. For a subset of the males (*n* = 15), we performed an additional trial at the end of the experiment, playing back the multimodal call stimulus once every four seconds (i.e. ‘slow multimodal call', figure 1) to control for the reduced call rate of the treatment in which the multimodal call alternated with a high-intensity swat ripple.

For each frog (*n* = 29 frogs; no. trials per frog: range = 1 to 5, median = 4; 106 trials total), we compared call rate (average no. calls per sec) and complexity (average no. chucks per whine) between control conditions (i.e. silent playback) and treatment playback. Males called during all silent control conditions (106/106 trials), but sometimes ceased calling (9/106 trials) or only produced a single call (2/106 trials) in response to a treatment playback. All 11 of these latter trials were excluded from call rate analysis, whereas only the nine trials in which the male ceased calling were excluded from complexity analysis. We also excluded two treatment playbacks due to playback error or a sudden, loud stimulus from the environment. All acoustic analyses were performed in Adobe Audition CS6.

### (b) Bat foraging and female choice experiments

The effectiveness and consequences of calling strategies are ultimately defined by the responses of the receivers. We therefore investigated how frog-biting midges impact decision-making in foraging bats and female túngara frogs by modulating the calling behaviours of male frogs and their rivals. Bats (*n* = 5; 10–20 trials per bat per experiment) and female túngara frogs (*n* = 24; 1 trial per female per experiment) were presented with two-choice tests of airborne (no ripples) frog call playbacks to assess preferences (see electronic supplementary material for detailed descriptions of methods).

To evaluate relative preferences between a calling, swatting male and a rival responding to his swat ripples (i.e. the scenario examined in the male behavioural experiment), we broadcast calls of a male who intermittently swats—i.e. representative of the call rate and complexity of a frog that swats every 2 s; 0.34 calls s^−1^, 1.00 chucks per call [19]—versus a calling rival male responding to high-intensity swat ripples (i.e. representative of the call rate and complexity of frogs that were exposed to a high-intensity swat ripple once every 2 s; 0.5 calls s^−1^, 1.75 chucks per call). In a second two-choice test, to investigate if and to what extent responding to swat ripples alters receiver preferences relative to a typical, non-swatting male, we broadcast calls of a characteristic non-swatting, calling male (i.e. representative of the call rate and complexity of a rival frog before being exposed to high-intensity swat ripples; 0.45 calls s^−1^, 1.00 chucks per call) and calling male responding to high-intensity swat ripples (same as in previously mentioned two-choice test). A third two-choice test was given to a single bat (*n* = 1; 20 trials), in which we broadcast calls of a male who intermittently swats, but does not append ornamental chucks (i.e. approximate call rate of a frog that swats every 2 s, but without ornamental chucks; 0.34 calls s^−1^, 0 chucks per call) versus a calling rival male responding to high-intensity swat ripples (same as above).

### (c) Statistical analyses

For our analyses, we formulated generalized linear mixed models fit under a Bayesian framework using the *brms* package version 2.14.4 [40] and R version 4.0.3 (R Core Team, 2020). We used weakly informative priors to ensure that draws from the prior could be from any hypothetical dataset [41]. In this way, these priors include probability mass around potential, but not implausible extreme values. We ran four chains with 3000 iterations each and discarded the first 500, resulting in 10 000 posterior samples. To ensure model convergence, we visually examined traceplots for good mixing of chains and confirmed that $\hat{R}$ < 1.1 for all parameters. We performed posterior predictive checks to assess model fit with the *bayesplot* package version 1.8.1 [42]. Models were compared by assessing expected log pointwise predictive densities with the *loo* package [43]. See electronic supplementary material, table S3 for details specific to each model. Effects were determined using the hypothesis function in *brms*, first with two-side tests to determine an effect and directionality and subsequently a *post hoc* one-sided test. Comparisons of effects between treatments were determined by subtracting the posterior probability densities from one another.

## 3. Results

### (a) Field recordings

Compared to call ripples, swat ripples were overall of greater maximum (swat *β* – call *β* = 1.94; 90% CI, 0.58 to 2.86; electronic supplementary material, figure S3) and root mean square (swat *β* – call *β* = 0.37; 90% CI, 0.09 to 0.67) velocity and dominant frequency (swat *β* – call *β* = 2.69; 90% CI, 0.55 to 4.80). Ripple types did not differ with regard to displacement measurements (electronic supplementary material). We confirmed call ripple displacement (median = 148.2; 95% CI, 104.4 to 215.3 µm max, peak-to-peak) was within the range previously reported [36].

The maximum velocity of swat ripples differed depending on whether the frog's swatting appendage faced towards or away from the point of the water surface being measured with the laser doppler vibrometer. When the swatting appendage was on the frog's side nearest to the laser, the swat ripples had a greater maximum velocity compared to when the swatting appendage was on the frog's side farthest from the laser (near *β* – far *β* = 3.01; 90% CI, 2.17 to 3.86; electronic supplementary material, figure S1). For playback calibrations, we set a rough threshold for ‘high' versus ‘low' categories of maximum velocity of swat ripple based on the median estimate from our model of swat ripples recorded in the field (5.84 mm s^−1^, 4.55–7.51 mm s^−1^ 95% CI).

### (b) Rival males

**Figure 2.**
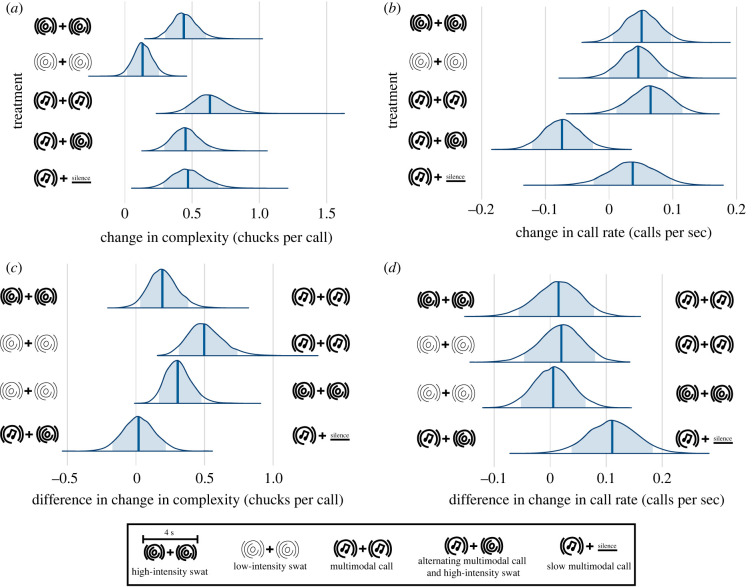
Calling responses of male túngara frogs to playbacks of mating calls and swats. Effect of playback treatments on (*a*) call complexity (no. chucks per call) and (*b*) call rate (no. calls per sec). Difference between treatment effects on (*c*) call complexity and (*d*) call rate, which were obtained by subtracting samples from posterior distributions between treatments in (*a*) and (*b*), respectively. Blue shading = 90% credible interval (CI); vertical line = median.

We predicted that rival male callers would respond similarly to both swat- and call-induced ripples, by increasing their complexity and call rate. There is strong evidence that males increased their average call complexity in response to all treatments (figure 2*a*; electronic supplementary material, figure S4 and table S4). Low-intensity swat ripple playback caused males to increase their complexity, although only slightly (*β* = 0.13; 90% CI, 0.02 to 0.25). High-intensity ripples elicited an increase of nearly 0.5 chucks per call (*β* = 0.44; 90% CI, 0.30 to 0.60), similar to the effect of multimodal calls alternating with high-intensity swats (*β* = 0.46; 90% CI, 0.29 to 0.64) and control slow multimodal call treatment (*β* = 0.48; 90% CI, 0.29 to 0.70).

Males responded with calls of greater complexity to high-intensity swat ripples than low-intensity swat ripples (high-intensity swat *β* – low-intensity swat *β* = 0.31; 90% CI, 0.17 to 0.48; figure 2*c*), while there was no difference in complexity in response to multimodal calls alternating with high-intensity swats compared to the control slow multimodal call treatment (control slow multimodal call treatment *β* – multimodal calls alternating with high-intensity swats *β* = 0.02; 90% CI, −0.17 to 0.22). Males, however, increased their complexity by over 0.5 chucks/call in response to multimodal calls (*β* = 0.64; 90% CI, 0.45 to 0.87). This effect was greater than either low- or high-intensity swat ripples (multimodal call *β* – low-intensity swat *β* = 0.51; 90% CI, 0.31 to 0.74; multimodal call *β* – high-intensity swat *β* = 0.20; 90% CI, 0.03 to 0.38).

There is also strong evidence that males slightly increased call rates in response to multimodal calls (*β* = 0.06; 90% CI, 0.01 to 0.12; figure 2*b*; electronic supplementary material, table S5). Males increased their call rates to a similar degree during playbacks of high- (*β* = 0.05; 90% CI, 0.01 to 0.10) and low-intensity swats (*β* = 0.05; 90% CI, 0.00 to 0.09; figure 2*c,d*). Males decreased their call rate in response to multimodal calls alternating with high-intensity swats (*β* = −0.07 ; 90% CI, −0.12 to −0.03). They did not, however, change their call rate in response to the control slow multimodal call treatment (*β* = 0.04 ; 90% CI, −0.02 to 0.10). When comparing these two treatments, there is strong evidence that multimodal calls alternating with high-intensity swats elicited reduced call rates in males compared to the control slow multimodal call treatment (control slow multimodal call treatment *β* – multimodal calls alternating with high-intensity swats *β* = 0.11; 90% CI, 0.04 to 0.18). Altogether, we find support for the prediction that swat-induced ripples elicit increased call complexity and rate in rival males.

### (c) Bat foraging and female choice

**Figure 3.**
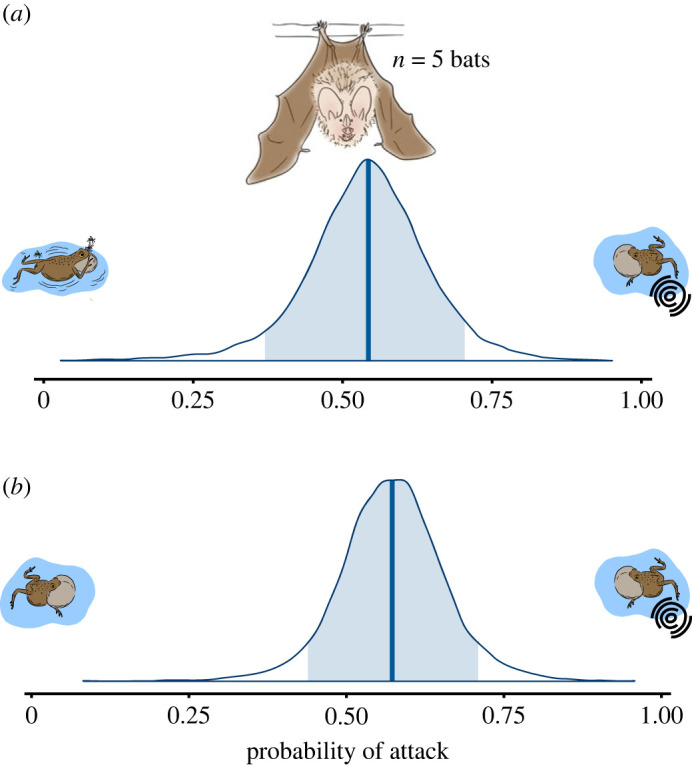
Probabilities of fringe-lipped bat attack in two-choice playback experiments. (*a*) Probability that a bat chooses a calling male who intermittently swats versus a calling rival male responding to high-intensity swat ripples. (*b*) Probability that a bat chooses a typical non-swatting, calling male and calling male responding to high-intensity swat ripples. In both panels, a probability of 0 indicates that a bat would always attack the playback with reduced call rate and complexity, whereas a probability of 1 indicates that a bat would always attack the playback with the increased call rate and complexity. 10–20 trials per bat per experiment.

Following the results from the rival male experiment, we tested the prediction that eavesdropping bats and female frogs would prefer the increased signal elaboration of the rival frog compared to the reduced signal elaboration of the swatting male. There was little to no evidence that bats prefer the rival male who responds to high-intensity swats when presented with a calling male that swats intermittently (probability of selecting rival male = 0.54; 95% CI, 0.32 to 0.74; *n* = 5; figure 3*a*) or a typically non-swatting, calling male (probability of selecting rival male = 0.57; 95% CI, 0.40 to 0.74; *n* = 5; figure 3*b*). Though purely an anecdotal observation, due to low sample size, one bat showed a consistent preference for the rival male when the focal male swatted intermittently while producing simple calls (i.e. without appended chucks; *n* = 1 bat, rival chosen in 19/20 trials).

**Figure 4.**
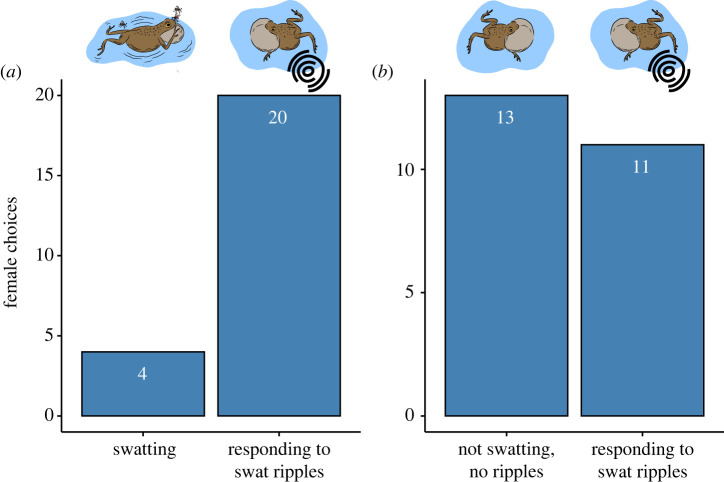
Mating choices by female túngara frogs. (*a*) Number of females who chose a calling male who intermittently swats versus a calling rival male responding to high-intensity swat ripples. (*b*) Number of females who chose a typical non-swatting, calling male versus a calling male responding to high-intensity swat ripples.

In contrast to bat preferences, there was strong evidence that females prefer rival males who respond to high-intensity swats when compared with calling males that intermittently swatted (probability of selecting rival male = 0.83; 95% CI, 0.65 to 0.94; *n* = 5; figure 4*a*). There was, however, little to no evidence that females selected rival males who respond to high-intensity swats compared to typically non-swatting, calling males (probability of selecting rival male = 0.46; 95% CI, 0.27 to 0.65; *n* = 5; figure 4*b*). That is, the change in the rival's calling behaviour in response to high-intensity swat ripples did not make the rival more attractive to females than he had been prior to receiving this stimulus. Rather, it was the relative difference between the rival's swat-elicited elaborated calling and the reduced elaboration of the swatting male that resulted in a female preference for the rival male.

## 4. Discussion

We find that ripples generated by the defensive swats of an attacked male cause his neighbouring rival to elaborate his call by increasing call rate and complexity. Thus, by eliciting anti-eavesdropper defences in their prey, the effects of eavesdropping frog-biting midges extend beyond the individual frog they attack to rivals in the chorus. At equivalent distances, swat ripples were greater than call ripples in both velocity and frequency, though the wide ranges of both ripple types overlapped. In natural conditions, males are exposed to this variation due to differences in the relative orientation of neighbouring swatting males. Natural fluctuations in ripple properties are also likely due to the heterogeneous environment at each chorus pond, in which barriers (e.g. vegetation, rocks, sticks) and water depth impact ripple transmission. This variable filtering can accentuate the ambiguity a receiver perceives between stimuli associated with distinct causes (e.g. call- versus swat-induced ripples), creating a difficult discrimination task for the receiver [44]. Despite being associated with distinct causes, we find that males elaborate their calls in response to both call- and swat-induced ripples. A similar effect is seen in males' generalized responses to call- and rain-induced ripples [33].

Though these frogs demonstrate a generalized response despite a ripple's underlying cause, a rival túngara frog's call effort diminishes the farther he is from the source of a call-induced ripple [32], suggesting that these frogs extract distance-based information from ripple characteristics which they use to tailor the magnitude of their response. Such information is important to signallers in lek mating systems, as they defend the space around them from competitors. While it is unclear which ripple characteristics are perceived by these frogs, several could impart distance-based information. First, a male may use the amplitude or velocity of the ripple wave, which attenuates due to spreading loss over distance. Consistent with a greater response to the ripple cues of a closer, more threatening competitor, we find that males increased their call complexity to a greater degree in response to high-velocity than low-velocity swat ripples (though there was no difference in call rates). Second, males might extract distance information from the relative intensities of the ripple frequencies. Higher frequencies attenuate more than low frequencies across distances, filtering the frequency profile of the ripple as it travels away from the source. Additionally, considering that the propagation velocities of surface waves are frequency-dependent, males may assess the temporal delay between the arrival of individual frequency components of the ripple. Those individual frequencies arrive at a receiver close in time at short distances from the ripple's origin, but exhibit greater delays as the distance increases [45,46]. If males only vary their response based on distance-based ripple properties, we would expect that rivals would elaborate their calls more in response to the higher frequency and velocity content of swat ripples than call ripples. Yet, males increased their call rate to the same degree regardless of ripple type. More puzzlingly, males increased their complexity more in response to call ripples than high-velocity swat ripples. Therefore, factors other than velocity appears to affect how ripples influence male calling behaviours.

While differences in frequency-based cues remain a possibility, multimodal integration can explain why males elaborated their complexity more to call ripples than swat ripples. For the call ripple treatment, the airborne call component was played back in synchrony with the call ripple, as occurs naturally. Call-induced ripples alter the calling behaviour of nearby rivals only when presented with the airborne call component [31]. This effect is not surprising given myriad studies demonstrating that male túngara frogs respond readily to airborne calls of competitors [23,38] and that multimodal integration enhances a receiver's response compared to the isolated effects of a stimulus's unimodal components [47,48]. The concomitant processing of ripple and airborne stimuli may be particularly important to males by providing redundant information when a unimodal component is masked by noise [47,49], or by reducing uncertainty in their perception of neighbour location and behaviour [44,50].

Irrespective of the mechanisms, rival males increased their complexity more to call ripples than high-velocity swats ripples, and more to high- than low-velocity swat ripples. Given that no single characteristic among these stimuli can account for this effect, it appears that males are attending to multiple features of the stimuli to inform call elaboration decisions. Future experiments that determine these causal mechanisms, and untangle their relative and interactive effects, are needed to fully understand the dynamic, complex interactions occurring within these social networks.

A particularly unexpected finding was that the alternating multimodal call and high-velocity swat ripple treatment resulted in the male reducing his call rate compared to the control slow multimodal call treatment. This outcome suggests that intermittent swatting reduces a rival's call rate. Why would this be if, at the same presentation rate, both playbacks with only swat ripples and with only calls increased call rates? One possibility is that the alternating ripple types affected the male's perception of the simulated rival, perhaps by disrupting his attention or overwhelming his sensory processing [47]. While we cannot rule out the possibility that males perceived the alternating multimodal call and high-velocity swat ripple treatment as a less competitive male, it is unlikely given that the treatment also caused the male to substantially increase call complexity to the same extent as the control slow multimodal call. Another intriguing possibility is that the rival perceives the alternating multimodal call and high-velocity swat ripple as a high risk of midge attack. The causal factor behind this result ultimately remains unknown. Clearly, however, while eliciting a generalized response in rival males, the sequential arrangement of call- and swat-induced ripples appears to modulate a rival's call elaboration.

Though sexual signals are a common target of eavesdropping enemies [1,2], little is known about the effects of these enemies beyond the target prey. Here, we find that an eavesdropping enemy indirectly elicits an elaborated sexual signal in the rival of its prey. To what extent this phenomenon extends across lekking anurans vulnerable to frog-biting midges, or lekking species in general, remains to be seen.

### (a) Bat foraging and female choice

Choice tests with females and bats suggest that the indirect, cascading effects of midge attacks may skew the reproductive opportunities away from the male defending himself from these eavesdroppers and towards the male's rival, while affecting neither male's risk of predation by bats. We found that, in responding to swat ripples, the increase in a rival's call rate and complexity does not make the rival more attractive to bats nor females than he was prior to swat ripple exposure. It is instead the concomitant shifts in calling behaviour—the prey reducing and the rival increasing call elaboration—that accounts for the relative increase in attractiveness of the rival male to females.

Our study is a first step towards understanding how eavesdropping micropredators, by affecting individuals beyond just their prey, can potentially warp the relative fitness of two neighbouring males in the chorus. Non-consumptive effects are appreciated in general predator–prey studies, but have received relatively little attention in the context of eavesdropping enemies and their signalling prey [2]. A future challenge resides in scaling up these interactions to the whole chorus while reflecting variation in midge attacks and male responses. Male call elaboration in the wild is driven in part based on rate of midge attack, which is highly variable [19]. In addition, rivals also swat in response to their own midge attacks generating feedback loops between neighbouring males. Swat ripples from the rival can thus propagate back to the original swatting male frog, ostensibly causing him to increase his calling behaviour amidst having to defend himself. Moreover, increased call rates and complexity can then attract more frog-biting midges [30]. It is unclear how these feedbacks alter the relative attractiveness of competing, swatting males to females, bats and frog-biting midges.

Male responses to midge attacks also vary based on how conservative or liberal each male's defensive swatting strategy is. While we found no difference in risk of bat attack between a typical swatting male and a male that responds to high-intensity swats, an anecdotal series of observations with a single bat leaves open the possibility that highly conservative males with less ornamented calls may shift nearly all predation risk on their rivals. Such conservative strategies in response to frog-biting midges are seen in nature [19] and bats prefer and better localize more ornamented calls [51]. Ultimately, the fitness landscape of males in a natural chorus is expected to be dynamic as it is modulated directly by biting midges and, as we show here, indirectly via associated effects from their neighbour's defensive swats. As micropredator eavesdroppers attacking frogs and toads are widespread with over 191 fly species across 23 genera recognized [52], and calling in water is common among anurans, this type of indirect interaction may be more ubiquitous than previously anticipated. Further work, however, is still needed to understand the nuances of these social interactions and how variable defensive strategies and frequencies of midge attacks interact with each other to impact the fitness outcomes of competing prey.

Our understanding of complex ecological systems is a product of the scale(s) at which we generate and evaluate hypotheses. To obtain a holistic view, one must assess the direct and indirect links between patterns and processes within and across scales of analysis [53]. Similarly, assessing the impact of eavesdropping enemies on prey behaviour and fitness in complex, multi-receiver systems should depend on the scale at which it is measured. Fine-scaled effects and their interactions may scale across social levels (e.g. individual < dyad < chorus) or may be lost as noise. For social contexts such as leks, in which each male interacts with nearby competitors, we predict that the eavesdropper-driven effects at the individual level and male-male dyad ‘ripple' throughout the social network, influencing calling behaviours beyond dyads up to the chorus.

We hypothesize that such eavesdropper-driven effects are likely influenced by the eavesdropping enemy's foraging strategy. Each consumer strategy is predicted to have unique effects on prey avoidance and defensive responses [54]. Whether different consumer strategies of eavesdropping enemies differentially impact the social interactions of their signalling prey remains an open question. We propose that micropredators lead to more nuanced, dynamic shifts in receiver behaviour at all scales given their frequency-dependent effects and lower total risk they exert per individual micropredator, compared to predators and parasitoids. Ultimately, this study highlights how and to what extent defensive strategies targeting eavesdropping enemies can indirectly modulate intrasexual competition and individual reproductive opportunities in a sexual communication system. By impacting female choice through a cascade of effects, eavesdropping micropredators (and likely other enemies) can exert nuanced constraints on the evolution of sexual signals and communication systems.

## Data Availability

All data and scripts associated with this manuscript are available on Dryad: https://doi.org/10.5061/dryad.z8w9ghxkd [37]. Additional information is provided in electronic supplementary material [55].
